# Prevalence and risk factors of latent tuberculosis infection among household contacts of tuberculosis patients: a cross-sectional study

**DOI:** 10.3389/fpubh.2026.1820601

**Published:** 2026-05-14

**Authors:** Pengxiang Huang, Tianli Ma, Bingqin Dai, Guoyu Huang, Lili Su, Shichao Shangguan, Mingxiao Yao, Cuixiao Liu, Shengyang Zhang

**Affiliations:** 1 Institute for Tuberculosis Prevention and Control, Shandong Center for Disease Control and Prevention, Jinan, Shandong, China; 2 Institute for Tuberculosis Control, Jinan Center for Disease Control and Prevention, Shandong University, Jinan, Shandong, China

**Keywords:** cross-sectional study, household contacts, latent tuberculosis infection, prevalence, risk factors

## Abstract

**Background:**

Household contacts (HHCs) of tuberculosis patients are at higher risk for latent tuberculosis infection (LTBI). However, current knowledge regarding LTBI prevalence and risk factors among HHCs in Shandong Province, China remains limited.

**Objective:**

This study aims to determine the prevalence of LTBI among HHCs of tuberculosis patients and identify associated risk factors.

**Methods:**

We conducted a cross-sectional survey among HHCs of tuberculosis patients in Shandong Province, China. LTBI prevalence was assessed using the tuberculin skin test (TST) and tuberculosis antigen-based skin test (TBST). Univariate and multivariable logistic regression analyses were performed to identify risk factors associated with infection.

**Results:**

A total of 5,772 HHCs of 1,657 tuberculosis patients were enrolled. The prevalence of LTBI among HHCs was 10.2% (95% CI: 9.424–10.985). In the multivariable logistic regression analysis, the infection rate increased in older age groups compared to the 0–14 age group. The highest adjusted odds ratio (aOR) was observed in the 45–59 age group (aOR = 3.761; 95% CI: 2.160–6.546). Living in multi-story buildings (aOR = 1.301; 95% CI: 1.024–1.651) and exposure to bacteriologically positive patients (aOR = 1.424; 95% CI: 1.066–1.903) were identified as potential risk factors for LTBI. In contrast, residing in a separate room within the same household was associated with a lower risk of infection compared to sharing the same room with the index patient (aOR = 0.608; 95% CI: 0.453–0.816).

**Conclusion:**

Households are high-risk settings for tuberculosis transmission. Therefore, it is necessary to intensify active contact screening at the household level to shorten the interval between tuberculosis diagnosis and contact tracing, thereby interrupting potential transmission chains within the community.

## 1 Introduction

Despite significant achievements in tuberculosis control over the past decade through enhanced patient detection and treatment management, resulting in a substantial decline in national incidence rates, China continues to bear a heavy burden of tuberculosis (1). According to the latest estimates from the World Health Organization (2), China remains one of the countries with a high tuberculosis burden. Additionally, it is estimated that approximately 350 million people in China are infected with *Mycobacterium tuberculosis* (MTB) (3). The population with latent tuberculosis infection (LTBI) serves as a vast reservoir for active tuberculosis, with approximately 5% to 10% of infected individuals progressing to active disease during their lifetime (4–6), thereby becoming new sources of tuberculosis. In 2015, the World Health Organization formally released its LTBI management guidelines, establishing systematic screening and preventive treatment for LTBI individuals as a key component of the three pillars of its tuberculosis elimination strategy and objectives (7).

Household contacts (HHCs) of tuberculosis patients are at high risk for LTBI. Studies indicate that HHCs of active tuberculosis patients face a higher risk of MTB infection and disease onset (8–10). With the accelerated aging of China's population and increasing population mobility, the risk of tuberculosis transmission within households, as the fundamental unit of society, may become more prominent. Tracing HHCs using the diagnosed patient as the index patient is a core component of the tuberculosis contact tracing strategy recommended by the WHO (7). In China, all tuberculosis patients are registered and managed at designated hospitals, a system that provides a solid operational foundation for the contact tracing strategy described above. Therefore, proactive screening and management of LTBI among HHCs of tuberculosis patients have become a critical component in breaking the chain of community transmission.

This study aimed to investigate potential risk factors for MTB infection among HHCs of tuberculosis patients. The findings provide evidence-based strategies to guide the management of HHCs in China.

## 2 Method

### 2.1 Study design

We conducted a cross-sectional study across 18 counties and districts in Shandong Province, China, from June 2023 to June 2024. We investigated and screened HHCs of tuberculosis patients. These tuberculosis patients initially presented and began receiving treatment at designated tuberculosis hospitals within the study area between June 2023 and June 2024. Patients were diagnosed with bacteriologically confirmed tuberculosis through sputum culture, smear, or nucleic acid amplification tests.

We defined HHCs as individuals who shared the same space with the index patient during their transmission period (from 3 months prior to diagnosis until 2 weeks after effective treatment initiation), with either continuous exposure of at least 8 h or cumulative exposure exceeding 40 h. Information on HHCs was obtained through self-report by the tuberculosis patient when they presented at the hospital, and subsequently verified by trained interviewers through face-to-face interviews with the contacts themselves. All individuals who met the definition of a household contact were included in this study, with no upper age limit. Written informed consent was obtained from all participants. For minor participants, in addition to obtaining written informed consent from their parents or guardians, we also obtained the child's assent where appropriate, based on their age and intellectual development.

We included HHCs who were able to complete the survey and infection testing at the study site and provided informed consent. Those who failed to complete the necessary interview, infection testing, or consent process were excluded from the study.

### 2.2 Data collection and tuberculosis infection detection

We screened HHCs through a combination of symptom assessment, chest X-ray, and either the tuberculin skin test (TST) or tuberculosis antigen-based skin test (TBST). According to the 2022, WHO consolidated guidelines on tuberculosis, both TST and TBST are recommended for the detection of tuberculosis infection (11). Both methods have demonstrated favorable screening performance in the Chinese population. In this study, the choice between TST and TBST was determined by each local health department based on local conditions, including reagent availability and staff training.

All HHCs aged 15 years and older were eligible for chest X-ray screening. For individuals younger than 15 years of age, chest X-ray was restricted to those with suggestive symptoms or a positive result on TST or TBST. Accordingly, age was categorized using 15 years as the cut-off to align with this screening protocol. All chest X-ray examinations and interpretations were performed at designated tuberculosis hospitals and were read by tuberculosis specialists with clinical experience. During the screening process, participants who presented with suggestive symptoms, abnormal chest X-ray findings, or a positive TST or TBST result received further confirmatory diagnostic testing for active tuberculosis at the same designated tuberculosis hospital.

Questionnaires were administered to HHCs through face-to-face interviews. Data collected included age, sex, education level, annual household income, physical activity, history of smoking and alcohol consumption, and living conditions. All questionnaire data were collected by staff who received standardized training.

### 2.3 Determination of LTBI

Considering screening costs and implementation feasibility, this study employed the TST or TBST as the detection method. TST uses purified protein derivative (PPD) as the antigen. It has the advantages of lower cost and simpler operation, but its specificity is reduced due to cross-reactivity with BCG vaccination. TBST uses a novel recombinant fusion protein containing only MTB-specific antigens, including early secretory antigenic target-6 (ESAT-6) and culture filtrate protein-10 (CFP-10). It is not affected by BCG vaccination and therefore offers higher specificity.

In this study, a positive TST indicating MTB infection was defined as an induration of ≥10 mm in individuals with a history of BCG vaccination, and an induration of >5 mm in those without a BCG vaccination history, who were HIV-positive, or who had received immunosuppressants for more than 1 month (12). Some regions in the study implemented the TBST. A positive result, defined as either erythema or induration ≥5 mm, was used to indicate MTB infection (12).

After excluding active tuberculosis, individuals with a positive TST or TBST result were classified as having LTBI.

### 2.4 Quality control

All field staff received standardized training and passed a competency assessment before the survey. TST and TBST procedures and result interpretations were strictly performed according to standard operating protocols by trained medical professionals. Induration and erythema diameters were measured using a transparent ruler to the nearest millimeter. Determination of MTB infection and pulmonary tuberculosis was conducted by physicians at designated tuberculosis hospitals. Data were double-entered and subjected to logical consistency checks.

### 2.5 Statistical analysis

Statistical analyses were performed using R (R Foundation for Statistical Computing, Vienna, Austria; version 4.5.2). Categorical data were described using frequencies and proportions. Univariate and multivariate logistic regression analyses were conducted to identify factors associated with LTBI among HHCs. Prior to multivariate logistic regression, variance inflation factors (VIF) were calculated to assess multicollinearity. All comparisons were two-sided, and *P* < 0.05 was considered statistically significant.

## 3 Results

### 3.1 Baseline demographic characteristics

A total of 5,772 HHCs were included in the analysis, with an average age of 47.2 ± 0.3 years. These contacts were traced from 1,657 tuberculosis patients, yielding an average of 3.5 HHCs per patient. Among the HHCs, 2,709 (46.9%) were male and 3,063 (53.1%) were female. For 80.1% of HHCs, the index tuberculosis patient they contacted was positive on at least one of the following testing methods: sputum smear, culture, or tuberculosis nucleic acid testing. A large proportion of HHCs had relatively low educational levels (70.2%), an annual income of less than 50,000 CNY (53.9%), or lived in a separate residence from the index patient (61.6%). The main characteristics of the study population are presented in Table 1. The prevalence of LTBI among all HHCs was estimated at 10.2% (95% CI: 9.424–10.985). Characteristics of HHCs with and without LTBI were compared, and significant differences were observed between the two groups, as shown in Table 1.

**Table 1:** Demographic and clinical characteristics of HHCs.

Variable	Total *n* (%)	LTBI		*χ^2^*	*P*-value
		Yes *n* (%)	No *n* (%)		
Total	5,772	589 (10.2)	5,183 (89.8)		
Age group (years)				18.224	0.003
0–14	535 (9.3)	30 (5.1)	505 (9.7)		
15–29	684 (11.9)	76 (12.9)	608 (11.7)		
30–44	1,138 (19.7)	133 (22.6)	1,005 (19.4)		
45–59	1,635 (28.3)	178 (30.2)	1,457 (28.1)		
60–74	1,336 (23.1)	135 (22.9)	1,201 (23.2)		
≥75	444 (7.7)	37 (6.3)	407 (7.9)		
Gender				0.090	0.765
Male	2,709 (46.9)	273 (46.4)	2,436 (47.0)		
Female	3,063 (53.1)	316 (53.6)	2,747 (53.0)		
Educational level				16.620	0.001
Junior high school or lower	4,049 (70.2)	375 (63.7)	3,674 (70.9)		
High school (Vocational school)	1,023 (17.7)	118 (20.0)	905 (17.5)		
Technical college/Associate degree	412 (7.1)	52 (8.8)	360 (6.9)		
Bachelor's degree or above	288 (5.0)	44 (7.5)	244 (4.7)		
Type of residence				28.627	0.000
Single-story rural house	3,509 (60.8)	298 (50.6)	3,211 (62.0)		
Multi-story building	2,263 (39.2)	291 (49.4)	1,972 (38.0)		
Living arrangement with the index patient				38.256	0.000
Shared the same room	788 (13.7)	115 (19.5)	673 (12.9)		
Separate room within the same household	1,428 (24.7)	178 (30.2)	1,250 (24.1)		
Different household	3,556 (61.6)	296 (50.3)	3,260 (63.0)		
Annual household income (CNY)				68.474	0.000
<50,000 (<7,000 USD^a^)	3,113 (53.9)	258 (43.8)	2,855 (55.1)		
50,000–90,000 (7,000–12,600 USD)	1,763 (30.6)	172 (29.2)	1,591 (30.7)		
≥90,000 (≥12,600 USD)	896 (15.5)	159 (27.0)	737 (14.2)		
Smoking				6.925	0.009
Yes	646 (11.2)	85 (14.4)	561 (10.82)		
No	5,126 (88.8)	504 (85.6)	4,622 (89.18)		
Current drinking				4.966	0.026
Yes	770 (13.3)	96 (16.3)	674 (13.0)		
No	5,002 (86.7)	493 (83.7)	4,509 (87.0)		
Physical exercise					
Never	1,630 (28.2)	175 (29.7)	1,455 (28.1)	0.083	0.959
Occasional	1,273 (22.1)	129 (21.9)	1,144 (22.1)		
Frequent	2,869 (49.7)	285 (48.4)	2,584 (49.8)		
Sleep duration				4.503	0.034
<8 h	4,054 (70.2)	436 (74.0)	3,618 (69.8)		
≥8 h	1,718 (29.8)	153 (26.0)	1,565 (30.2)		
Self-rated sleep quality				10.825	0.001
Poor	213 (3.7)	36 (6.1)	177 (3.4)		
Fair	5,559 (96.3)	553 (93.9)	5,006 (96.6)		
History of tuberculosis				3.345	0.067
No	5,722 (99.1)	580 (98.5)	5,142 (99.2)		
Yes	50 (0.9)	9 (1.5)	41 (0.8)		
Knowledge of tuberculosis transmission routes				0.022	0.882
Yes	5,421 (93.9)	554 (94.1)	4,867 (93.9)		
No	351 (6.1)	35 (5.9)	316 (6.1)		
Bacteriological status of the household index patient				19.130	0.000
Negative	1,148 (19.9)	77 (13.1)	1,071 (20.7)		
Positive	4,624 (80.1)	512 (86.9)	4,112 (79.3)		
Treatment history of the household index patient				1.690	0.194
New patient	5,549 (96.1)	572 (97.1)	4,977 (96.0)		
Retreatment patient	223 (3.9)	17 (2.9)	206 (4.0)		

a USD equivalents are based on the 2023 average exchange rate of 1 USD to 7.0 CNY.

### 3.2 LTBI associated factors: multivariable logistic regression

The results of multivariate logistic analyses are shown in Table 2. In the multivariable logistic regression analysis, after adjusting for covariates, the infection rate increased in older age groups compared to the 0–14 age group. The highest adjusted odds ratio (aOR) was observed in the 45–59 age group (aOR = 3.761; 95% CI: 2.160–6.546). Regarding type of residence, HHCs living in multi-story buildings had a significantly higher risk of infection compared to those living in single-story rural houses (aOR = 1.301; 95% CI: 1.024–1.651). Compared with HHCs who shared the same room with the index tuberculosis patient, those residing in a separate room within the same household had a lower risk of infection (aOR = 0.608; 95% CI: 0.453–0.816). Analysis of socioeconomic factors showed that HHCs with higher annual household income had a significantly higher risk of infection compared to those with the lowest household income (aOR = 2.239; 95% CI: 1.673–2.996). In addition, HHCs exposed to tuberculosis patients with positive bacteriological results had a significantly higher risk of infection than those exposed to bacteriologically negative patients (aOR = 1.424; 95% CI: 1.066–1.903). The VIF values for all independent variables in the model are less than 1.5, indicating no multicollinearity exists among the independent variables.

**Table 2:** Multivariate analysis of factors associated with LTBI.

Variable	Unadjusted model			Adjusted model		
	cOR	95%CI	*P*-value	aOR	95%CI	*P*-value
Age group (years)						
0–14	1.000			1.000		
15–29	2.104	1.357–3.263	0.001	3.109	1.726–5.601	0.000
30–44	2.228	1.478–3.358	0.000	3.297	1.847–5.884	0.000
45–59	2.057	1.379–3.067	0.000	3.761	2.160–6.546	0.000
60–74	1.892	1.257–2.848	0.002	3.477	1.944–6.219	0.000
≥75	1.530	0.929–2.520	0.095	2.950	1.454–5.986	0.003
Gender						
Male	1.000			1.000		
Female	1.026	0.865–1.218	0.765	0.940	0.744–1.187	0.940
Educational level						
Junior high school or lower	1.000			1.000		
High school (Vocational school)	1.277	1.026–1.591	0.029	1.106	0.835–1.465	0.484
Technical college/Associate degree	1.415	1.038–1.929	0.028	0.887	0.591–1.330	0.562
Bachelor's degree or above	1.767	1.260–2.478	0.001	1.270	0.835–1.931	0.263
Type of residence						
Single-story rural house	1.000			1.000		
Multi-story building	1.590	1.340–1.887	0.000	1.301	1.024–1.651	0.031
Living arrangement with the index patient						
Shared the same room	1.000			1.000		
Separate room within the same household	0.833	0.647–1.073	0.157	1.064	0.773–1.466	0.702
Different household	0.531	0.422–0.669	0.000	0.608	0.453–0.816	0.001
Annual household income (CNY)						
<50,000 (<7,000 USD^a^)	1.000			1.000		
50,000–90,000 (7,000–12,600 USD)	1.196	0.977–1.465	0.083	1.277	0.983–1.658	0.067
≥90,000 (≥12,600 USD)	2.387	1.928–2.956	0.000	2.239	1.673–2.996	0.000
Smoking						
Yes	1.000			1.000		
No	0.720	0.563–0.920	0.009	0.979	0.682–1.407	0.910
Current drinking						
Yes	1.000			1.000		
No	0.768	0.608–0.969	0.026	0.839	0.569–1.237	0.376
Physical exercise						
Never	1.000			1.000		
Occasional	0.968	0.761–1.231	0.790	0.903	0.705–1.156	0.418
Frequent	0.004	0.762–1.298	0.966	0.992	0.751–1.312	0.956
Sleep duration						
<8 h	1.000			1.000		
≥8 h	0.811	0.669–0.984	0.034	1.107	0.860–1.424	0.430
Self-rated sleep quality						
Poor	1.000			1.000		
Fair	0.543	0.376–0.785	0.001	0.647	0.382–1.098	0.106
History of tuberculosis						
No	1.000			1.000		
Yes	1.946	0.941–4.024	0.072	2.223	0.935–5.285	0.071
Knowledge of tuberculosis transmission routes						
Yes	1.000			1.000		
No	0.973	0.679–1.395	0.882	0.963	0.564–1.642	0.889
Bacteriological status of the household index patient						
Negative	1.000			1.000		
Positive	1.732	1.350–2.221	0.000	1.424	1.066–1.903	0.017
Treatment history of the household index patient						
New patient	1.000			1.000		
Retreatment patient	0.718	0.435–1.186	0.196	0.870	0.474–1.597	0.653

a USD equivalents are based on the 2023 average exchange rate of 1 USD to 7.0 CNY.

## 4 Discussion

HHCs of tuberculosis patients are the population closest to the infection source and at the highest risk of tuberculosis infection. This group represents a priority target for tuberculosis screening and intervention. Implementing effective prevention and control measures for this population is of significant importance in reducing the incidence of tuberculosis. We estimated the prevalence of LTBI among HHCs of registered tuberculosis patients undergoing treatment to be 10.2% (95% CI: 9.424–10.985) based on skin test screening. It is noteworthy that this finding is lower than the infection rates reported in similar studies conducted in other regions of China (29.6%−39.1%) (13, 14). This discrepancy may be attributed to multiple factors. In this study, screening of HHCs was initiated concurrently with patient identification, inevitably leading to an underestimation of the LTBI prevalence due to the time lag involved in developing adaptive immune responses to MTB (15).

Furthermore, differences in screening methods may represent another important factor contributing to the disparity in tuberculosis infection rates among HHCs. The screening method used in this study was the tuberculosis infection skin test, whereas studies reporting higher infection rates more frequently employed IFN-γ release assays (IGRA). IGRA with higher specificity exhibits greater sensitivity (16, 17), but it requires peripheral blood sampling and carries higher testing costs, making it difficult to implement comprehensively in large-scale screening programs.

In addition, the positivity rates of TST and TBST may differ within the same population. Although both tests rely on the diameter of induration or erythema for interpretation, TBST may exhibit higher specificity in BCG-vaccinated populations due to differences in antigen components and immune response mechanisms, thereby reducing false-positive rates (18). Furthermore, the administration and interpretation of skin tests are subject to operator variability. Differences in training levels and technical skills across regions and healthcare facilities may affect the stability and comparability of positivity rates. Nevertheless, an infection rate of 10.2% still underscores the high risk of tuberculosis infection among HHCs, and systematic screening and management of this population hold clear public health value. In future studies, simultaneous comparison of TST, TBST, and IGRA in screening practice may help clarify the impact of methodological differences on results. At the same time, it is recommended that initially screen-negative individuals undergo at least 12 months of active follow-up to minimize missed diagnoses due to delayed immune responses. Establishing standardized training, certification, and blind re-reading mechanisms for skin test operators can also minimize operational bias.

In this study, increasing age was identified as a risk factor for HHCs developing LTBI. Consistent with previous studies (19, 20). In many tuberculosis high-burden countries, standard tuberculosis prevention practices include administering a single dose of BCG vaccine at birth. Although the BCG vaccine is effective in preventing miliary and meningeal tuberculosis in infants and children, its protective efficacy against tuberculosis in adults remains controversial (15, 21). With increasing age, the waning protective efficacy of the vaccine and the cumulative risk of MTB exposure (22) make it difficult for contacts exposed to a high-tuberculosis environment at home to clear MTB infection, thereby further increasing the risk of MTB infection in the host. In our study, type of residence was also identified as a key risk factor for MTB infection among HHCs. Specifically, contacts residing in multi-story buildings exhibited a significantly higher risk of infection than those living in single-story houses. Furthermore, compared to sharing a room with a tuberculosis patient, contacts living in completely separate houses had a significantly lower risk of infection. Multiple studies have indicated (14, 22, 23) that shared housing and overcrowding are typical risk factors for tuberculosis, as they increase exposure to infectious patients and thereby facilitate transmission within the same household. Sharing a room with the source of infection represents a high-intensity, continuous exposure and thus poses the greatest risk. Compared to single-story detached houses, multi-story buildings are associated with relatively limited living space and poorer ventilation conditions. Family members may spend more time and interact more frequently within confined functional spaces, thereby increasing opportunities for contact. Therefore, in public health guidance practices, it is crucial to educate patient households on reducing indoor exposure concentration and proximity by improving the indoor environment.

In addition, our study also found that HHCs exposed to patients with positive bacteriological test results had a higher risk of MTB infection. This finding is consistent with relevant epidemiological evidence (23–25). Patients with positive bacteriological results are generally considered to have relatively high transmissibility (26, 27). This may be attributed to the higher bacterial load shed by bacteriologically positive patients (28), which exposes HHCs in close daily contact to a long-term, high-density bacterial environment, thereby increasing the risk of MTB infection.

Notably, this study observed an increased risk of infection among HHCs with higher household income. Tuberculosis has traditionally been closely associated with poverty. However, with economic development and urbanization, the pattern of infection risk may become more complex in specific regions or population contexts. One possible explanation is that higher income may be associated with potential risk environments and behaviors, such as living in more enclosed high-rise apartments and engaging in more intensive indoor family interactions. Within this framework, household income may be linked to income-specific exposure patterns rather than the economic factor itself.

This study has limitations. First, due to cost considerations, TST or TBST was used as the screening method for LTBI. Although these are widely used standard methods, their results may be influenced by prior BCG vaccination or infection with non-tuberculous mycobacteria. Second, we relied on self-reported risk factor information from HHCs. We were unable to assess the potential impact of underlying reporting bias or recall bias on the results. In particular, variables such as smoking and drinking may be subject to social desirability bias and underreporting. Moreover, these variables were collected only as binary indicators (yes/no) without dose information. Third, the duration of exposure was not recorded or evaluated. Particularly among HHCs, exposure duration is a critical factor influencing the risk of LTBI. Fourth, this study only included HHCs of tuberculosis patients, and the findings cannot be directly extrapolated to other high-risk populations for LTBI, such as healthcare workers or people living with HIV.

## 5 Conclusion

In summary, this study focused on HHCs of tuberculosis patients and implemented a community-based active screening program for LTBI. This study represents a significant exploration of proactive screening strategies for tuberculosis high-risk populations. This study preliminarily determined the LTBI infection rate among HHCs of tuberculosis patients in this region and identified key factors associated with infection risk. These findings provide valuable insights for enhancing prevention and control measures targeting this high-risk group of HHCs of tuberculosis patients.

## Data Availability

The original contributions presented in the study are included in the article/supplementary material, further inquiries can be directed to the corresponding authors.
